# Gender Differences in Postural Stability among 13-Year-Old Alpine Skiers

**DOI:** 10.3390/ijerph17113859

**Published:** 2020-05-29

**Authors:** Agnieszka D. Jastrzębska

**Affiliations:** Department of Physiology and Biochemistry, University of Physical Education, 51-612 Wroclaw, Poland; agnieszka.jastrzebska@awf.wroc.pl

**Keywords:** body sway, Wingate test, adolescents, alpine skiers, postural control, gender, fatigue

## Abstract

This experiment examined changes in body sway after Wingate test (WAnT) in 19 adolescents practicing alpine skiing, subjected to the same type of training load for 4–5 years (10 girls and nine boys). The postural examinations were performed with eyes open (EO), eyes closed (EC), and sway reverenced vision (SRV) in the medial-lateral (ML) and anterior-posterior (AP) planes. The displacement of center of foot pressure (CoP), range of sway (RS), mean sway velocity (MV), way length, and surface area were measured in bipedal upright stance before and after the WAnT to assess the influence of fatigue on postural balance. There were no significant differences in WAnT parameters between girls and boys. Relative peak power (RPP), relative total work (RWtot) were (girls vs. boys) 8.89 ± 0.70 vs. 9.57 ± 1.22 W/kg, *p* < 0.05 and 227.91 ± 14.98 vs. 243.22 ± 30.24 W/kg, *p* < 0.05 respectively. The fatigue index (FI) was also on similar level in both genders; however, blood lactate concentration (BLa) was significantly higher in boys (10.35 ± 1.16 mM) than in girls (8.67 ± 1.35 mM) *p* = 0.007. In the EO examination, statistically significant differences between resting and fatigue conditions in the whole group and after the division into girls and boys were found. In fatigue conditions, significant gender differences were noted for measurements in the ML plane (sway path and RS) and RS in the AP plane. Comparison of the three conditions shows differences between EO vs. EC and SRV in AP plane measured parameters, and for RS in ML plane in rest condition in girls. The strong correlations between FI and CoP parameters mainly in ML plane in the whole group for all examination conditions were noted. By genders, mainly RS in ML plane strongly correlates with FI (r > 0.7). No correlation was found between BLa and CoP parameters (*p* > 0.06). The presented results indicate that subjecting adolescents of both genders to the same training may reduce gender differences in the postural balance ability at rest but not in fatigue conditions and that girls are significantly superior in postural balance in the ML plane than boys. It was also shown that too little or too much information may be destructive to postural balance in young adolescents.

## 1. Introduction

The ability to maintain body balance, either static or dynamic, is of great importance for performing everyday activities, as well as for the development and optimization of an athlete’s basic motor skills [1,2]. It is also one the most important skills that protect from injury. It has been established that highly trained athletes show more accurate postural control than sedentary subjects [3,4], and differences in postural control become noticeable between trained and untrained adolescents [5,6]. Non-athletes show lower postural control, mainly in the mediolateral plane compared to 13-year-old soccer players [5] and greater CoP path length and velocity compared to young karate athletes [6]. In addition to overall improvement of postural stability, the training process also affects the development of specific optimal postural strategies for a given discipline [7,8,9,10,11,12,13]. 

In alpine skiers, postural performance, among muscle strength, coordination, and anaerobic and aerobic performance, seems to play a fundamental role [14,15,16]. This is due to a combination of factors that, in and of themselves, threaten postural stability, and altogether raise the possibility of serious injury. During downhill skiing, the skier has to keep static posture toward the skis, as well as continuously striving to maintain the balance of the entire “man–skis” relative to the ground. Thus, postural performance may have a strong effect on postural control during sliding. Rapidly changing surrounding and surface textures causes an increase of somatic, vestibular, and sight information in the assessment of what happens with the body in a space. Ski training also improves the ability of situation-dependent changes in muscle recruitment [16]. Permanent displacement of the center of gravity of the body, caused by body twisting, uneven terrain, and the quality of the ground, causes the involvement of muscle, joint, and skin mechanoreceptors in the pursuit of maintaining a dynamic body balance [17]. Changes in acceleration, both angular and linear, increase the share of vestibular information in posture control when skiing, and the continuous shift in space increases the afferent impulses from the sight [18]. When skiing, the image recorded by eyesight changes constantly; therefore, sight provides important information related to the kinematics of body displacement, speed, and direction [19]. This mixed sensory information reaches the central nervous system, which is responsible for integrative approach and formation of appropriate muscle synergy to maintain equilibrium [20].

The time of development of individual senses and their joint integration was already a topic of research [21,22,23,24,25,26]. The influence of training on the postural control of children and young adolescents in some sports has also been recognized [6,27,28]. However, despite the importance of postural balance ability in alpine skiers, young adolescents have not received adequate attention in this field. There is a gap in knowledge about postural balance changes as a result of ski training and how fatigue induced by short, intensive effort may influence postural sway in young skiers. Researchers are mainly focused on the physiological aspects of skiing, like muscle strength [14,15] and aerobic and anaerobic performance [13,29,30,31,32]. What is more, the examined groups of skiers are older adolescent or adults and are most often elite athletes [14,32,33]. In the context of young adolescent skiers, the literature is even poorer and mainly concerns risk factors causing injuries during alpine skiing [34,35]. Astonishingly, available data are more epidemiological than in term of postural balance control importance in injury prevention. Identification of ski-specific risk factors for injuries in youth skiers is lacking. The only study related to this topic treats poor physical fitness of youth skiers as an injury risk factor [36]. This may be due to the fact that children’s balance abilities continue to develop up to late adolescence [37]. As balance is important for movement skills development, proper movement execution during downhill skiing in rapidly changing conditions, and injury prevention, it was decided to assess the postural stability of youth alpine skiers in resting and fatigue conditions.

The purpose of the presented study was to assess the influence of acute fatigue caused by the Wingate test (WAnT) on balance control in youth skiers. WAnT is a supramaximal 30-s cycling test for anaerobic performance assessment. Its validity and reliability have been proven in the examination of various sport groups, in sedentary subjects, and in children [38,39]. The WAnT was chosen as the acute fatigue that causes is characteristic for slalom giant, which takes youth competitors around 35–45 s. Both slalom giant and WAnT cause a deep disturbance of homeostasis as the amounts of anaerobic contribution in both is high, and in the laboratory tests are even higher than during downhill skiing [32]. It was assumed that out of vision condition (EC) will not cause the decrease of maintenance of body balance and that visual feedback will have a positive effect on body balance in both rest and post-exercise conditions. The second purpose was to examine the gender differences in youths’ body balance control after an intensive short effort as well as show gender differences in postural control in adolescent skiers. According to previous reports suggesting that from age 3-4 years girls exhibit superior postural stability [40,41,42,43], it was hypothesized that young adolescent girls have superior postural stability than boys, both in rest and fatigue conditions. 

## 2. Materials and Methods

The study involved 19 adolescents (10 girls and nine boys; mean age: 13.50 ± 1.31) practicing alpine skiing. All examined participants were students of the primary Winter Sports Championship School. The full characteristics of the group can be seen in Table 1. Participants were trained in school for 4–5 years and in the last three years had 3–4 2-h training sessions per week. All participants were free of known balance disorders, neuromusculoskeletal impairments, and injury history for the last year. Three days prior to the examination, the participants were free of any organized activity (training session or physical education classes).

The participants were blind about the aim of the investigation. Informed written consent was obtained from all subjects, their parents, and their couch after the testing protocols had been explained. The study was approved by the local ethics commission and was in accordance with ethical aspects of the Declaration of Helsinki. All measurements took place in a laboratory with internal control of temperature and humidity (21 °C and 55%, respectively). The examinations were conducted between 11:00 and 15:00, as anaerobic performance increases with time of day [44] and the circadian rhythms have less influence on static postural control [45,46].

**Figure 1:**
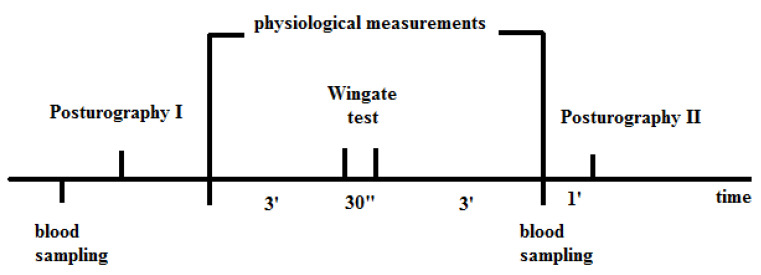
Scheme of visit in the laboratory.

To identify the effects of acute fatigue on postural stability in children, the maximal 30-s Wingate test (WAnT) was engaged (Figure 1). In order to exam postural stability, a Posturograph XY force platform (Olton, Warsaw, Poland) fitted with four strain gauges (one in each corner) with dedicated software was used. The sensors measured displacement of center of foot pressure (CoP) on platform in frontal—medio-lateral (ML) and sagittal—anterio-posterior (AP) directions. Data were collected and analyzed using the software supplied with the platform, which continuously records the CoP trajectories at a sampling rate of 100 Hz. Measurements were performed in three different conditions—eyes open (EO), eyes closed (EC), and sway referenced vision (SRV). During SRV trial participants were watching the CoP marker movements, displayed on a screen situated 3 m in front of them on head level. Each trial took 32 s. Before trials, the children had 30 s brake to relax. The dependent variables of postural balance measurement were CoP sway area (SA [mm^2^]), sway path length (SP [mm]), and mean velocity of CoP displacement (MV [mm/s] and range of sway (RS [mm]). All parameters were assessed for total CoP medio-lateral (ML) and anterio-posterior displacement (AP). During the examination, the participants stood upright, barefooted, two-legged, and wearing comfortable clothes. They were instructed to stand on the platform with arms hanging loosely at the side. Their feet were positioned in a triangle shape (heels 3 cm apart, at an angle 20° between the medial side of feet) corrected by the examiner, according to the standardization process for the platform. One day prior to the test, a visit to the laboratory and practice run were allowed to ensure that subjects felt comfortably in laboratory area. 

WAnT was performed on a mechanically braked by the friction belt cycle ergometer Monark 895E (Monark, Vansbro, Sweden) with 7.5% of participant body mass braking force, which was originally proposed for children [47]. The seat height was adjusted individually so that the angle of deflection was no greater than five degrees when the leg was fully extended. Before the test, the children undertook standardized warm-up on a cycle ergometer Monark 895E for 5 min with the last 60 s interspersed with two all-out sprints of 5 s. After 3 min of passive recovery, they completed the 30-s test. Each child was instructed to pedal as fast as possible from the beginning and maintain maximal speed for 30 s. The verbal encouragement was given to each participant. After termination of the test participants worked slowly without resistance for the next three minutes. In the fifth minute of recovery, the participant stepped on the platform in order to assess post-effort stability. 

Maximum (Pmax) and minimum anaerobic power (Pmin) were defined as the highest and the lowest mechanical powers, recorded over a 0.5-s period. Index fatigue (IF) was calculated as a difference between Pmax and Pmin and expressed as percentage of Pmax.

Capillary blood samples (10 μm) were collected from the fingertips for blood lactate concentration (BLa) before warm-up and in third minute of recovery. BLa concentrations were determined using photometer LT400 (dr Lange, Berlin, Germany). 

For body composition measurement, a near infrared device NIR (F6100/XL, Futrex, Hagerstown, MD, USA) was used. This device allows the assessment of fat percentage (PF%), fat mass (FM), and free fat mass (FFM). The device measures the optical density of body tissues on the biceps brachii of the dominant hand to estimate the PF %. It is a commonly used method for body composition measurements [48,49,50]. Following the suggestion of Freedman et al. [51], fat mass and free fat mass were standardized for height^2^. FMI was calculated as fat mass (kg)/height^2^ and FFMI as free fat mass (kg)/height^2^. 

Statistica 13 Software (StatSoft Inc., Tulsa, OK, USA) was used for statistical analysis. All results are expressed as mean ± SD (standard deviation). Data normality was assessed through the Shapiro–Wilk *W*-test. Once the assumption of normality was confirmed the parametric tests were involved. Student’s paired *t* test was performed to test significance between pre- and post-effort trials, between girls (G) and boys (B) in pre- and post-effort condition separately, and between AP and ML plane for girls and boys in pre and post effort condition. One-way analysis of variance (ANOVA) with a sub-sequent Tukey post-hoc test (if differences between groups were revealed) were performed to examine differences between stabilography parameters among the three examined conditions. The Pearson correlation was calculated for WAnT and posturographic parameters. The level of statistical significance was set at *p* < 0.05. 

## 3. Results

### 3.1. Anthropometric and WAnT Results

Both girls and boys were similar with regard to their anthropometric characteristics (Table 1). Neither body mass (*p* = 0.66), nor height (*p* = 0.11), nor BMI (*p* = 0.25) varied between both groups, whereas body composition significantly differed between two groups in absolute (Table 1) and standardized to highest fat mass index (FMI; *p* < 0.000) and free fat mass index (FFMI; *p* < 0.000).

WAnT test results are presented in Table 2. There is no statistically significant difference between girls and boys in parameters describing their anaerobic performance except for time to peak power (TP*_peak_*) (*p* < 0.05). However, it can be seen, that boys achieved higher absolute and relative peak power (PP, RPP) and total work (W*_tot_*, RW*_tot_*) than girls. The blood lactate concentration (ΔBLa) after the WAnT was significantly higher in boys (10.35 ± 1.16 mM) than girls (8.67 ± 1.35 mM) *p* < 0.01. No significant correlation was found between BLa and posturographic parameters in general and by gender.

The only WAnT parameter correlated on significant level with CoP parameters is fatigue index (FI). In the whole examined group in EO condition, a strong correlation was found between FI and all measured CoP parameters, with the exception of surface area, with correlation coefficient ranging between 0.5 and 0.7 (*p* < 0.05). In EC condition, the strong correlation was found only for parameters in ML plane (SP r = 0.569, *p* = 0.02; MV r = 0.523, *p* = 0.04; RS r = 0.625, *p* = 0.03) and in SRV condition only for RS (r = 0.663, *p* = 0.01) in ML plane.

By gender, in boys correlation coefficients on a very high level were noted for RS in ML (r = 0.796, *p* = 0.03) and AP plane (r = 0.720, *p* = 0.07) in EO, and for RS in ML plane (r = 0.786, *p* = 0.02) in EC condition. In girls, the only correlations found were for RS in ML plane in EO (r = 0. 551, *p* = 0.12) and SRV condition (r = 0.532, *p* = 0.17), but at an insignificant level.

### 3.2. Sway Control

#### 3.2.1. Influence of Gender Factor on Body Sway 

The possible influence of gender on postural control was examined by comparing the results of posturography balance measurement in both pre- and post-effort conditions in three examined conditions. There is no gender-effect on body sway in pre-effort condition for EO and EC. In SRV, there were noticed differences between girs and boys for ML plane parameters: SP and MV (*p* < 0.05). In post-effort conditions, gender-related differences were noted for all three conditions and are mainly related to the ML plane (EO: SP and MV *p* < 0.01; EC: SP and RS *p* < 0.05; SRV: SP and MV *p* < 0.05, and RS *p* < 0.01) and in the AP plane to the RS (EC *p* < 0.05 and SRV *p* < 0.05) (Table 3). The results show greater sway control by girls in post-effort condition. 

#### 3.2.2. Influence of Effort on Body Sway

Larger differences between rest and post-exercise tests were recorded in boys. While in the rest conditions boys were more stable than girls, after exercise, the differences between the groups decreased as a result of smaller changes in postural balance in girls and greater changes in boys. Statistically significant differences were noticed in EO examination for all parameters independently on gender. EC examination shows less stability for boys, which is confirmed by significant increase in SA (*p* < 0.05), MV-AP (*p* < 0.01), RS-AP (*p* < 0.01), RS-ML (*p* < 0.05) parameters. In visual feedback (SRV), conditions effort did not influence statistically postural body sway in neither girls nor boys (Table 3). 

#### 3.2.3. Effect of Condition on Body Sway

Stability was assessed in three conditions—eyes open (EO), eyes closed (EC), and sway referenced vision (SRV). The vision effect was significant in girls before the WAnT for SP (F = 7.65, *p* < 0.01), MV (F = 8.60, *p* < 0.001) and in the AP plane for SP (F = 12.80, *p* < 0.000), MV (F = 13.69, *p* < 0.000) and RS (F = 11.97, *p* < 0.000). A closer look (Table 3) revealed that this effect was mainly due to a contrast between EO vs. EC conditions. There was no effect of test conditions in boys. 

## 4. Discussion

This study explored gender differences in postural stability and influence of fatigue induced by WAnT on postural stability in adolescent skiers. The parameters used in this study for body balance assessment are based on CoP displacement. CoP displacement provides a quantitative differentiation for static balance examination [52]. The hypothesis that girls have superior postural stability than boys, both in rest and fatigue condition have been partially supported by obtained results. The results obtained during pre-test examination did not confirm the hypothesis, but the results obtained after the WAnT are consistent with it. 

The obtained results show slight gender differences in postural balance during standstill at rest. Sporadically, significant differences among girls and boys noted in rest conditions for SP and MV in ML plane for SRV conditions may be attributed to boys’ attentiveness. Under the SRV condition, focused attention on the control of body sway increases neuromuscular activity [53]. It is likely that boys’ worse postural balance control may be due the lower attentiveness and concentration as well as lower integrity of sensory information by the central nervous system [43,53]. Results by Vando et al. [54] show improvement of stability in young karate athletes after visual feedback training. Perhaps adding this type of training in young skiers would improve their attentiveness and consequently their postural control mechanism based on visual feedback during downhill skiing, which could potentially decrease injury risk. However, this requires further research.

There is consensus in the literature that children aged 5–10 years old show gender differences with superior postural balance control by girls independently on test performed (one-leg or tandem standing) [37,40,41,42,43,55,56,57,58,59]. It is also known that postural sway decreases linearly with age [37,42,55]. Additionally, it was observed that body sway in double leg quiet stance decreases with age in boys but not in girls, and at age 10, no significant gender differences were noted [39]. Our results show that young adolescents aged 13–14 years generally do not exhibit gender differences during standstill in comfort and rest condition what is consistent with longitudinal study conducted by Rachner et al. [58]. Interestingly, the same authors found the differences between girls and boys in postural balance control in 14- to 16-year-old adolescents. These gender differences may be due the vestibular system maturation. Between 7–10 years of age, girls’ vestibular system may mature earlier than boys’ [60]. On the other hand, with a rapid growth spurt during puberty, some decrease in postural control may be seen due to changes in physique or hormonal balance [61]. The conflicting results and opinions show, that there is no clear age-related moment when balance development reaches a similar level among both genders. Moreover, in trained children, the training process can overlap with morphological development, causing specific adaptation changes resulting from the performed activity. In our research, adolescent were subjected to the same type of training load for a long time (4–5 years), and the anthropometrical measurements (Table 1) showed the homogeneity of the whole examined group regarding body mass, height, and BMI. It is likely that the lack of gender differences in the ability to maintain balance in rest condition is here the result of training adaptation. The improvement of postural control in children, with its sport specificity, is recognized, while the reduction of gender differences as a result of the same training has not yet been demonstrated.

The presented study revealed that EC and SRV conditions affected postural balance in sagittal (AP) plane in girls and in frontal (ML) plane in boys. Concomitantly, the girls demonstrated larger CoP excursion in AP plane and the boys in ML plane, in both rest and post WAnT conditions. The results obtained by the girls are consistent with those noted by Odenrick and Sandstedt [37]. However, those differences between body sway in sagittal and mediolateral direction were described for both genders [37], and our results demonstrate that boys have higher sway amplitude in mediolateral than in sagittal direction. These findings did not confirm the hypothesis that out-of-vision condition (EC) does not influence the stability of youth skiers. The lack of vision negatively influenced the postural balance of examined girls at rest at a significant level. When vision is suppressed, greater participation of other senses is engaged in postural balance control. It was reported that girls’ vestibular system maturate earlier than in boys [59]. Considering this, it would be expected that girls show less body sway impairment than boys when vision is suppressed. The present results indicate the need of examination of young athletes to better understand the physiological mechanisms of balance control in active youths. Extended knowledge in this area can be used for creating new balance training programs for young athletes to improve their physical ability and prevent a risk of injuries.

Moreover, SRV condition did not reveal a positive effect on postural stability. It was expected that during the sway referenced vision trial (SRV), the improvement of postural sway would be noticed. During SRV, participants were to keep the dot symbolizing CoP displayed on the monitor situated in front of them unmoving. The results obtained during SRV are between values measured in EO and EC condition, and in the case of boys, in the ML plane, are even worse than in EC. Lack of improvement of postural stability in focusing on the dot enhanced attention (SRV) could be explained by immaturity of visual afferents in examined adolescents in postural balance control [21]. On the other hand, skiers use vision for sighting the rapidly changing environment and not postural processing. It seems that too little (EC) or too much (SRV) information in alpine skier adolescents affects their postural control. Presumably, this is related to the immaturity of nervous system [24,26] and the still low level of integrative possibilities [62]. On the other hand, these results may indicate specific adaptation of young skiers. They preferentially use vestibular or proprioceptive information to control postural stability as vision is used for sighting. 

The results presented in this study demonstrate that short anaerobic effort provides fatigue, which affects postural balance in adolescents. As we were interested in acute fatigue induction and its impact on postural balance in ski trained adolescents and not strictly anaerobic performance estimation, in our study, we used WAnT from the battery of tests estimating anaerobic capacity [63]. Its character causes deep disturbance of homeostasis [64], similar to those obtained after a ski race [32]. It was also reported that fatigue induced by strenuous anaerobic exercise leads to balance deficits that can cause injuries [65]. The acute fatigue disturbs postural balance by degrading the effectiveness of both sense input and motor output what interrupt postural functions [66]. 

We did not find any studies comparing changes in body balance caused by short maximum anaerobic effort among the girls and boys. Poor evidence in this topic also applies to adults. We have found some information how anaerobic effort (of various character) influences the postural balance in adults, however with no gender division [9,67]. Obtained results also showed that scale of it depends on gender. We noted a decrease in postural stability after the WAnT in both groups, however, the magnitude of changes vary among girls and boys. In girls, a smaller effect of performed WAnT on body balance was noticed than that in boys. The sway area increased in girls about 35% (*p* < 0.05) and 20% (insignificant) for EO and EC conditions, respectively, while in boys, the increase in sway area was of 75% (*p* < 0.05) and 45%, respectively (*p* < 0.05). Boys also show lower stability than girls for almost all parameters in ML plane and for RS in AP plane (Table 3). Greater postural stability in girls may be attributed to more advanced neuromuscular development [68], maturation level of vestibular system [59], or more improved sensorial integration [21] than in boys. It was also reported that boys comparing to girls in age under 10 years are less attentive [21]. It is likely that boys aged 13.5 years still shows hyperactivity, which is responsible for the maturational slowness in boys [59].

The other finding of the presented study are significant gender differences in postural stability in the ML plane for EO, EC, and SRV trials (Table 3). Analyzing differences between girls and boys in post exercise condition it was noticed that in ML plane girls showed significantly greater postural control than boys regardless of the test conditions (EO, EC, SRV). Interestingly, boys show significantly lower visual feedback capacity in maintenance of stable posture in both pre- and post-test conditions (*p* < 0.05). Similar levels and directions of changes were recorded for range of sway (RS) in EC and SRV condition in AP plane (*p* < 0.05). An explanation for this phenomenon could be seen in anthropometric factors [69]—somehow, examined young adolescents were rather homogenous anthropometrically (Table 1). The significant gender differences noted in the presented results (Table 1) could translate into slightly higher results obtained in the WAnT in boys than girls on an insignificant level (Table 2) and higher acidosis in boys (10.35 ± 1.16 mM vs. 8.67 ± 1.35 mM; *p* < 0.01). That, in turn could, translate to greater posture maintenance disruption. Nevertheless, the anthropometric parameters and their relation to postural balance changes resulted by anaerobic effort has not been directly tested and requires further investigation in adolescent alpine skiers.

Despite the decrease in postural stability after WAnT, no correlation was found between acidosis level, WAnT parameters except for index of fatigue (IF), and CoP displacement parameters. BLa concentration after WAnT informs the contribution of glycolysis in the production of energy for muscle work; however, it must be remembered that its concentration in blood is balanced by rate of production and removal. The lack of acidosis correlation with the correlation of IF with CoP displacement parameters, observed mainly in the ML plane, can indicate the nervous origin of postural balance control impairment in examined young adolescents in fatigue conditions. These results correspond to other findings [66], which stated that postural control in the ML direction is substantially related to hip and knee muscles fatigue but not ankle muscles. In WAnT, the main working muscles are the hip and knee flexors and extensors. Gribble and Hertel [66] stated that the fatigue of these muscles causes impairments of postural control. Additionally, slowed conduction of afferent signals from the fatigued muscles may lead to slowed propagation of efferent signals [62], which in turn may be manifested in a higher postural sway expressed by an increase of the sway path and mean velocity of CoP excursion (Table 3). Further research should be conducted to highlight this phenomenon. It should be pointed here that all examined participants were strictly positioned on the balance platform, so the position had no influence on presented result. There were also no visible differences in body stature among girls and boys. 

The limitation of presented study is a small group subjected to research. It is difficult to have access to youth athletes trained in same, discipline-specific way. The discipline trained by participants is also a limitation here, as skiing on sport level is not popular in Poland. In some cases we did not get permission for blood sampling, in other for such strenuous effort as WAnT. The other limitation is lack of control group. Here I would pay attention to the character of used methods for fatigue development. Untrained subjects in age of 13 yr are not prepared to perform such exhaustive effort as WAnT. Even trained children, somewhat accustomed to this type of effort, lose consciousness after the test. Six of participating young athletes did not finish postural control examination after they performed WAnT. Also, blood collection for non-trained children to satisfy curiosity is rather unethical. Some kind of limitation was also less detailed anthropological measurements. However, correlation between anthropological data, effort reaction, and postural balance control were not the aim of presented study, this information would be helpful in an explanation of our obtained results. 

## 5. Conclusions

This study extends the literature in the area of young adolescents’ exercise physiology in term of adaptive changes in body balance caused by regular ski training, as well as how single maximal anaerobic effort affects the postural balance control in youth ski racers. Presented results show that (a) postural reaction on short-term, intensive exercise and its character and size depends on gender and (b) the quality and quantity of information has an impact on posture maintenance ability; too little or too much information may be destructive to body balance in early adolescence. 

Conducting the postural measurements in non-fatigue and fatigue conditions potentially highlights neuromuscular and postural control deficits after an anaerobic maximal effort. As balance skill is a possible factor affecting injuries during skiing, its deficit in fatigue condition may increase the risk of injury. Awareness of these changes should direct the attention, both researchers’ and coaches’, to the postural abilities of young skiers. Skier injuries in youth can reduce the willingness to participate in this form of activity and cause interruption of a sport career.

## Figures and Tables

**Table 1:** Physical characteristics of examined group (mean ± SD, t, *p*).

	Avg ± Std	Girls *N* = 10	Boys *N* = 9	t	*p*
Age [years]	13.50 ± 1.31	13.86 ± 1.00	13.05 ± 1.55	1.410	0.176
Body mass [kg]	54.05 ± 6.78	53.42 ± 6.32	54.82 ± 7.61	−0.450	0.658
Height [cm]	163.45 ± 7.85	160.91 ± 6.20	166.56 ± 8.86	−1.675	0.111
BMI [kg·m^−2^]	20.19 ± 1.49	20.42 ± 1.23	19.68 ± 1.50	1.191	0.249
FM [kg]	8.58 ± 4.25	11.83 ± 2.46 *	4.60 ± 1.73	7.433	0.000
FFM [kg]	45.42 ± 6.92	41.58 ± 3.87 *	50.11 ± 7.07	−3.432	0.003
PF [%]	15.92 ± 7.35	21.94 ± 2.32 *	8.57 ± 3.29	10.649	0.000
FMI	3.26 ± 1.62	4.54 ± 0.76 *	1.68 ± 0.69	8.710	0.000
FFMI	16.91 ± 1.34	16.03 ± 0.73 *	17.98 ± 1.01	−4.687	0.000

BMI—body mass index, FM—fat mass, FFM—free fat mass, PF—percent fat in body mass, * *p* < 0.05 for the difference between groups.

**Table 2:** Results of Wingate test (mean ± SD, t, *p*).

Kolumna1	Avg ± Std	Girls *N* = 10	Boys *N* = 9	t	*p*
PP [W]	502.79 ± 84.35	477.09 ± 59.85	526.00 ± 103.51	−1.312	0.206
RPP [W·kg^−1^]	9.22 ± 1.01	8.89 ± 0.70	9.57 ± 1.22	−1.509	0.149
W*_tot_* [kJ]	12.77 ± 2.11	12.15 ± 1.44	13.33 ± 2.78	−1.229	0..235
RW*_tot_* [J·kg^−1^]	234.21 ± 23.19	227.91 ± 14.98	243.22 ± 30.24	−1.554	0.138
FI	15.42 ± 5.45	14.64 ± 4.08	16.50 ± 7.09	−0.726	0.447
TP*_peak_* [s]	6.61 ± 1.21	7.05 ± 1.31*	6.01 ± 0.77	2.117	0.049
T*_m_* [s]	4.87 ± 1.77	4.80 ± 1.39	4.84 ± 2.36	−0.055	0.957

PP—peak power, RPP—relative peak power, W*_tot_*—total work, RW*_tot_*—relative total work, FI—fatigue index, TP*_peak_*—time to achieve peak power, T*m*—time of maintaining peak power, * *p* < 0.05 for the difference between groups.

**Table 3:** Postural stability parameters (mean (SD) in rest and after WAnT condition for girls and boys. Results for *Student t* test for intersex comparison and for effort influence.

			Rest		Post		Rest	Post	G	B
			G	B	G	B	G vs. B	G vs. B	r/p	r/p
Area/s [mm^2^]		EO	319 (157)	270 (173)	436 (200)	471 (173)			0.011	0.028
		EC	464 (209)	414 (189)	575 (.36)	596 (202)				0.042
		SRV	329 (131)	319 (197)	350 (185)	314 (211)				
Way/s [mm]		EO	247 (52)	249 (059)	306 (68)	376 (56)		0.054 ns	0.009	0.001
		EC	335 (63)	339 (132)	377 (87)	434 (109)				
		SRV	316 (54)	349 (127)	323 (66)	373 (120)				
Mean velocity [mm/s]		EO	7.6 (1.6)	7.8 (1.6)	9.6 (2.2)	11.1 (2.0)			0.01	0.001
		EC	10.4 (1.8)	11.6 (4.6)	11.9 (2.6)	13.6 (3.4)				
		SRV	9.91 (1.6)	10.7 (4.0)	10.2 (1.9)	11.5 (3.7)				
Anterior-Posterior	Way/s [mm]	EO	167 (44)	156 (30)	203 (43)	238 (47)			0.004	0.001
		EC	250 (57)	227 (103)	274 (50)	290 (77)				
		SRV	218 (38)	195 (68)	222 (48)	219 (87)				
	Mean velocity [mm/s]	EO	5.4 (1.4)	4.7 (1.0)	6.2 (1.3)	7.1 (1.3)			0.037	0.001
		EC	7.8 (1.8)	6.6 (2.1)	8.7 (1.6)	9.1 (2.4)			0.052 ns	0.002
		SRV	6.9 (1.0)	6.1 (2.4)	6.9 (1.5)	6.8 (2.6)				
	Range of sway [mm]	EO	20.4 (7.5)	21.7 (5.7)	28.2 (5.4)	33.4 (7.5)			0.0004	0.004
		EC	31.5 (8.6)	27.2 (8.9)	34.0 (3.7)	38.6 (4.4)		0.048		0.005
		SRV	30.7 (3.3)	29.4 (6.2)	31.8 (3.5)	37.3 (5.3)		0.038		0.013
Medial-Lateral	Way/s [cm]	EO	138 (31)	171 (45)	178 (48)	251 (47)		0.011	0.003	0.004
		EC	178 (47)	252 (136)	193 (48)	277 (85)		0.032		
		SRV	173 (48)	255 (117)	181 (51)	279 (84)	0.047	0.018		
	Mean velocity [mm/s]	EO	4.5 (0.8)	5.3 (1.6)	5.4 (1.7)	7.1 (2.1)		0.011	0.023	0.003
		EC	5.7 (1.5)	7.9 (4.0)	6.3 (1.9)	8.6 (2.6)		0.059 ns		
		SRV	5.5 (1.4)	8.0 (3.6)	5.9 (1.5)	8.8 (2.5)	0.048	0.017		
	Range of sway [mm]	EO	22.1 (7.7)	22.9 (8.1)	29.1 (6.9)	32.6 (7.2)			0.0004	0.003
		EC	30.7 (7.6)	28.9 (9.6)	30.1 (6.4)	38.4 (6.8)		0.031		0.018
		SRV	28.4 (5.5)	34.7 (10.1)	28.6 (5.4)	37.8 (5.5)	0.086 ns	0.008		

EO—eyes opened, EC—eyes closed, SRV—sway reverenced vision. Statistical significance set on *p* < 0.05 level.
